# Relationship between socio-economic characteristics of older adults’ women and family planning use in Botswana

**DOI:** 10.1080/17290376.2020.1858945

**Published:** 2021-02-28

**Authors:** Njoku Ola Ama, John O. Olaomi

**Affiliations:** a Department of Statistics, University of Botswana, Gaborone, Botswana; b Department of Statistics, University of South Africa, Florida Campus, Roodepoort, Johannesburg

**Keywords:** Family planning, older adults, correlation, multinomial logistic regression

## Abstract

Older adults (50 years and over) are still sexually active and therefore vulnerable to unplanned pregnancy, infection of STIs and HIV, yet there are no programmes in place to cater for their family planning needs. The objective of the study is to show how some socio-economic characteristics of older adults influence their family planning (FP) use. The study used a stratified random sampling design where four health districts (two urban and two rural) were purposively selected and the sample size of 444 older adult women allocated to the districts using proportional allocation to size. Snowball technique was used in identifying respondents. The multinomial logistic regression analysis reveals that while age, marital status, educational qualification, employment status, menopausal status, district and desire for another child jointly significantly predict FP use, only menopausal status and desire for another child individually significantly (*p* < 0.01) predict FP use. Older adult women who desired another child were significantly (*p* < 0.01) 7.5 times more likely to use family planning (FP) methods than those who do not want another child. The postmenopausal older adult women were less likely to use FP methods than those in their premenopausal state (OR = 0.13). Women with no schooling were less likely to use FP methods than those with degree/professional qualifications. Single and married women were less likely to use FP methods than the divorced/widowed/separated. The study recommends the promotion of education and training on FP use among the older adult women that will take into consideration their menopausal status and desire for another child. The training should be home-based.

## Introduction

A lot of efforts and resources have been put in by International Organisations and national governments to improve the sexual and reproductive health (SRH) including family planning seeking behaviours of women of sexual active ages 15–49 years, and those of their children (Schäferhoff, van Hoog, Martinez, Fewer, & Yamey, 2019). However, there are no programmes that have been put in place to promote the quality of life and meet the family planning needs of the older adults (50 years and above). Yet the older adults (50 years and over) have family planning needs either to stop/delay pregnancies or prevent HIV and STI infections. Family planning represents one of the four pillars, besides antenatal care, safe delivery and postnatal care used as a strategic public health intervention to reduce maternal mortality in developing countries where 99% of global maternal deaths occur (Ahmed, Li, Liu, & Tsui, 2012). The older adults engage in risky sexual behaviours thinking that they can no longer be pregnant and may be less likely to use condoms during sex than their younger counterparts (Lindau et al., 2007; Stall & Catania, 1994). Although quite a substantial number of the women, 50 years and over, may have gone beyond their reproductive life span, yet they are sexually active (Dennerstein, Dudley, & Burger, 2001; Reddish, 2002). The use of family planning services (e.g. condom) for those who might have stopped bearing children could be to help them to avoid contracting STIs, and HIV and AIDS, thereby improving the quality of their lives, while at the same time continuing to enjoy their sexual relationships. Data on Family Planning needs, STI or HIV infection rates do not usually include over-50s, despite the fact that older men and women continue to engage in sexual relations well into old age. It is important, therefore, that services for STI and counselling regarding safe sex should be available to persons of all ages. Additionally, understanding how some key sociodemographic variables, namely, age of woman, marital status, employment status, highest educational level, health district of women are from, menopausal status, and desire for another child affect the family planning use of older adult women is important in guiding formulation of appropriate programmes and interventions to improve the quality of life of the older adults.

Studies by OlaOlorun (2013), Holden et al. (2005), Janus and Janus (1993), Patel, Gillespie, and Foxman (2003), Dennerstein et al. (2001) and Reddish (2002) have shown that women as well as men, 50 years and over, have been sexually active. Janus and Janus (1993) reported that the sexual activity of women remains relatively stable as they move from young adulthood through mid-life and into the postmenopausal phase, with over two-thirds of women aged 39–50 (68%) and 51–64 (65%) reporting engaging in sexual intercourse at least once a week. A study of women aged 18–94 by Patel et al. (2003) found similar frequency of intercourse through age 59. While decreased libido has been reported as one menopause-related change (Dennerstein, Alexander, & Kotz, 2003; Reddish, 2002), other women reported an increase in sexual desire during and after menopause (Koster, 1991; Koster & Garde, 1993). From 1992 to 1998, Xu, Schillinger, Aubin, St Louis, and Markowitz (2001) reported that there were 1535 episodes of STIs for the 50- to 80-year-olds in Washington State, accounting for 1.3% of all reported STIs. Tuddenham, Page, Chaulk, Lobe, and Ghanem (2017) found that the overall prevalence of acute sexually transmitted infections was 18.1% (CI 0.17–0.19) in older adults in Baltimore, Maryland with Older women more likely to be diagnosed with trichomoniasis (21.5% [CI 18.6–24.5]). Thus, there are a lot of concerns on the health and welfare of older adults that should attract the attention of public health managers and policy makers.

It is worth recognising that the older adults (50 years and over) represent a significant section of the population and should be seen and acknowledged by society as active agents of societal development. They help in achieving truly transformative, inclusive and sustainable development outcomes in their different countries. Older persons, particularly older women, provide unpaid care for spouses, grandchildren and other relatives, including those with disabilities (UNFPA and HelpAge International, 2012). Grandparents, especially older adult women, have become central and indispensable to the well-being of families, in the HIV/AIDS pandemic and growing migration to look for greener pastures by parents, especially in the absence of public care and other social services (Ama, 2011). Studies have shown that 69 percent of Bolivian migrants who moved to Spain left their children at home, usually with grandparents; in rural China, grandparents look after 38 percent of small children whose parents had gone to cities for work; and in some parts of Colombia, around a third of internally displaced older persons were responsible for caring for grandchildren (UNDP, 2016; UNFPA and HelpAge International, 2012).

It is crucial to the health and well-being of older women, children and adolescents that unintended pregnancies be prevented, and adolescent childbearing reduced through universal access to family planning services. In 2017, 78 per cent of women of reproductive age (15–49 years of age) worldwide who were married or in union had their need for family planning satisfied with modern methods, up from 75 per cent in 2000. Progress has been substantial in the least developed countries, with a rise of 18 percentage points from 2000 to 2017 (UNDESA, 2017), but nothing is being done to ameliorate the plight of the older adults’ women. As the elderly population is likely to increase in the future in Botswana as in other countries, and there is a definite shift in the disease pattern, i.e. from communicable to non-communicable, it is high time that the health care system gears itself to the growing health needs of the older adults in an optimal and comprehensive manner. Although the Government of Botswana has introduced the old age pension scheme through the Ministry of Local Government (Republic of Botswana, 2020), as a measure to improve the lifestyle and well-being of the older adults aged 65 years and above, this policy does not include health care benefits, such as provision of free medical attention to the older adults including family planning services.

This paper will (i) analyse, at the individual level, menopausal status and desire for another child by older adults, together with a number of demographic factors such as age of woman, marital status, employment status, highest educational level and district, associated with the use of family planning services by older adults in Botswana and (ii) determine how these characteristics influence the family planning use. It is hoped that the findings of this study will assist the Government of Botswana to reposition the national family planning programme in the country to cater for the older adult women.

## Theoretical framework

The conceptualisation of this paper has been influenced by a set of behavioural theories: the Health Belief Model (HBM) and the Theory of Reasoned Action. The Health Belief Model is based on the principle that the likelihood of someone adopting preventive behaviour is influenced by two factors: the perceived risk of a disease and the perception that the benefits of adopting the behaviour outweigh the costs of doing so (Fishbein & Guinan, 1996; Strecher & Rosenstock, 1997). Thus, in this paper, the use of family planning is influenced by their desire to save them from the risk involved in further pregnancy at this older age or contraction of STIs and HIV/AIDS. The Theory of Reasoned Action, on the other hand, states that a person’s intentions are the best guide to behaviour. The intentions are further determined by his or her attitude towards the behaviour and the subjective norm (Fishbein & Guinan, 1996). In this paper, older adults’ intention or desire to use the family planning to either prevent further pregnancies, for those who have reached their desired fertility levels but are still in relationship and are sexually active, is to prevent HIV and STI infections. The perceived risks and intention to adopt family planning method can be influenced by socio-economic and demographic characteristics of the older adults, namely: age, marital status, educational qualification, employment status and locality, want to have another child and menopausal status, among many others (see Kposowa, 2013; Yihunie, Reda, Tamene, Benedict, & Deribe, 2013 and Arbab, Bener, & Abdulmalik, 2011).

## Methods

*Design:* The study used a cross-sectional and survey design, interviewing older adults to collect data on individual, social network and organisation level factors, sexual activities and needs, family planning needs and limitations, any forms of bias or stigma in accessing family planning services. Four health districts, two urban (Gaborone and Selibe Phikwe) and two rural (Barolong and Kweneng East) were purposively selected for the study because of their closeness to each other in terms of location (two in the south and two north east) to reduce cost of the survey. The choice of equal number of urban and rural health districts was to assist comparison of urban and rural women’s experiences on family planning health issues. The stratified random sampling design, with the four selected health districts, Gaborone, Selibe Phikwe, Barolong and Kweneng East (Rural) as strata, was employed (see also Ama & Ngome, 2013). The study population was all women, 50 years and above, from the four selected districts. The 2001 projected populations of the older adult women (50 years and above) from the selected sites (Central Statistics Office, 2011) were 7, 408 for Gaborone; 1, 983 for Selibe Phikwe; 3,381 for Barolong and 9,676 for Kweneng East. This gave the total target population as 22,448 older adult women.

*Sample size*: A statistically acceptable sample size of 454 was determined for the study using the Creative Research System (2012), a sample size calculator, at 95% confidence level allowing an error margin of 5%. This sample size of 454 (which includes adjustment for people in the original sample who may refuse to participate in the study) was allocated to the selected districts using proportional allocation to size. At the end of the data collection, a total of 444 older adult women responded in completing the questionnaire. This gave a response rate of 98%.

*Identification of respondents*: The snowball technique (Singh, Pandey, & Aggarwal, 2007) was used in identifying women in the selected districts because of the sparse nature of the population and the difficulty in obtaining an updated sampling frame of older adult women.

*Instrument for the study and data collection*: The questionnaire was used in the study for data collection. The questionnaire contained questions on the socioeconomic and demographic characteristics of the older adult women, family planning activities and use. The questionnaire was piloted at Tlokweng on a population similar to the one being studied (women 50 years and above) for content, ambiguity, clarity, time for interview and administration of questionnaire before being used for the study. A two-day training workshop for the research assistants was organised and the contents of questionnaires were explained to them. The questionnaire was administered to the respondents by the trained research assistants who met the respondents in their homes or at work. The Setswana version (translated version of the questionnaire) was used in the case of those who could not understand English. The research assistants explained the purpose of the study to the respondents; assured them of confidentiality of information provided and that the questionnaire did not contain any identification of respondents; and informed them that participation was not compulsory and there was no payment for participation. Those willing to participate signed a consent form before the interview began.

*Ethical clearance*: The instrument was reviewed by the University of Botswana ethical committee, the Ministry of Health Research and Ethical Committee and permission to conduct the study obtained from the District Health Management Teams in the studied health districts before being used.

*Data analysis*: Data were captured using the Statistical Programme for Social Sciences (SPSS) version 24 and analysed using descriptive statistics (percentages, correlation coefficient) and inferential statistics (chi-square). The Multinomial Logistic Regression Model was fitted to the data and the Likelihood Ratio test statistics used in testing for the significance of the regression coefficients.

## Results

### Socio-demographic characteristics

Over half of the women (53.2%) were between 50 and 59 years while 27% were between 60 and 69 years. Only 13.5% of the older adult women were between 70 and 79 years and 6.3% were 80 years and above. 42.8% of the women had no educational qualification (no schooling) while 26.4% had primary school certificate. A little more than 24% had either had secondary school certificate/Diploma while 6.8% had university degree/professional certificate. A little more than two in every three women (68%) were unemployed while 32% were employed. Most of the women (32.9%) were married while 27.9% were single (never married); 24.1% were widowed, 6.3% were cohabiting and 8.6% were divorced. The women who desired another child were 3.4% while 25.2% were currently using FP methods to prevent future pregnancy (Table 1).

**Table 1.** Characteristics of sampled older adults’ women (see also Ama & Olaomi, 2019).

Characteristics of older adult women sampled		Frequency	Percent
Age of women	50–59	236	53.2
	60–69	120	27.0
	70–79	60	13.5
	80 and above	28	6.3
Employment status	Unemployed	302	68.0
	Employed	142	32.0
Highest Educational qualification	No schooling	190	42.8
	Primary Certificate	117	26.4
	Secondary Certificate/Diploma	107	24.1
	University Degree/Professional Certificate	30	6.8
District	Gaborone	150	33.8
	Kweneng East	207	46.6
	Selibe Phikwe	45	10.1
	Barolong	42	9.5
Marital Status	Never married (Single)	124	27.9
	Cohabiting	28	6.3
	Married	146	32.9
	Divorced	38	8.6
	Widowed	107	24.1
	Separated	1	0.2
Desire another child	Yes	15	3.4
	No	415	93.5
	Don't know	14	3.1
Using methods to prevent future pregnancy	Yes	112	25.2
	No	316	71.2
	Don't know	16	3.6
Have you attained menopause	Yes	383	86.3
	No	61	13.7

### Older adult women’s attitude towards sex and sexual activities

Table 2 shows the general attitude of the older adult women to sex and sexual activities. The responses show that 35% of the women still enjoy and have sex with their partners while 21% of them wished they could refrain themselves from the urge to have sex and 13% took a renewed interest in sex after attaining menopause. To majority of them (75%), better health for themselves and their partners is all they need most while two in every three women (67%) do not consider sexual activities as important to them now. While 61% of the older adult women considered emotional closeness as equally or more important than the physical satisfaction, 48% of them think that finding a partner is the most important factor toward a better sexual life.

**Table 2.** Attitude of older adult women to sex and sexual activities (N=355).

General attitude of older adult women to sex and sexual activity		
Better health for me or my partner is all I need most	265	75%
Sexual activity is not important to me now	237	67%
Emotional closeness is equally or more important than the physical satisfaction	218	61%
Finding a partner is the most important factor toward a better sexual life	171	48%
I still enjoy sex with my partner	123	35%
Less stress and more time are the top things that would improve my sexual life	97	27%
My partner has to fondle me to get me aroused for sexual activity	83	23%
I wish I could refrain myself from the urge to have sex	74	21%
Sexual intercourse is uncomfortable (dyspareunia)	64	18%
Sexual intercourse is painful	49	14%
I took a renewed interest in sex after menopause	46	13%
I feel guilty because of inability to meet my partner’s sexual needs	38	11%
I feel rejected and undesirable because I cannot have sex with my partner	24	7%
My partner suffers from erectile dysfunction (ED)	13	4%

### Correlation between family planning use and socio-demographic characteristics

The use of family planning methods to prevent future pregnancy/STIs is significantly positively correlated with desire for another child (*r* = 0.205, *p* < 0.01), age of the women (*r = *0.162, *p* < 0.01), educational status (*r* = 0.104, *p* < 0.05) and marital status (*r* = 0.055). It is significantly negatively correlated with employment status (*r* = −0.088, *p* < 0.05), locality (District of origin) (*r* = - 0.106, *p* < 0.05), and menopausal status (*r* = - 0.302, *p* < 0.01) (Table 3).

**Table 3.** Correlation matrix between use of FP methods and dependent variables.

		Using methods to prevent future pregnancy	Age of women	Meno-pausal status	District	Marital status	Employ-ment status	Educa-tional status	Desired anotherchild
Using methods to prevent future pregnancy /STIs	Correlation Coefficient	1	0.162**	−0.302**	−0.106*	0.055	−0.088*	0.104*	0.205**
	Sig. (1-tailed)	.	0.000	0.000	0.014	0.128	0.035	0.016	0.000
	N	428	428	428	428	428	428	428	427

The relationship between older adults’ use of family planning to prevent pregnancy and menopausal status, desired another child, age, marital status, educational status, employment status, district of the respondents was established by fitting the multinomial logistic regression (Starkweather & Moske, n.d.; Schwab, 2002) model to the data with outcome (dependent) variable as ‘Use family planning to prevent further pregnancy’. The independent and dependent variables are coded as shown in Table 4.

**Table 4.** Description of Variables for Multinomial Logistic Regression Analysis (see also Ama & Olaomi, 2019).

Dependent variable	Use FP to prevent pregnancy	1= Yes; 2= No; 3 = Don’t know
Independent Variables	Age (completed years)	1=50-59; 2=60-69; 3=70 and above
	Marital Status	1=Never married (Single); 2= Married; 3= Other
	Employment Status	1 = Unemployed; 2 = Employed
	Educational Status	1= No schooling; 2 = Primary Certificate; 3 = Secondary Certificate; 4 = Diploma/ Degree
	Desired another child	1=Yes; 2 =No;
	District (Location)	1 = Urban; 2 = Rural
	Menopausal Status	1 =Postmenopausal; 2 =Premenopausal

The Likelihood Ratio Tests were used to test the adequacy of the fitted model (Chi-square = 61.171, df = 11 and *p* <0.01), implying that the sociodemographic variables jointly predicts family planning use by the older adults (Table 5).

**Table 5.** Test of the overall fit of the Logistic Regression Model.

Model Fitting Information				
Model	Model Fitting Criteria	Likelihood Ratio Tests		
	−2 Log Likelihood	Chi-Square	Df	Sig.
Intercept Only	291.19			
Final	230.02	61.171	11	0.000

The result of the test of significance of the individual parameters in the model (Table 6) shows that the variables: desired another child and menopausal status of the older adult women highly significantly (*p* < 0.05) predict the use of family planning method holding other variables constant. However, location, educational qualification and marital status, employment status and age of the women are not significant factors (*p* > 0.05), Thus the main determinants of the older adults’ use of FP methods are desire to have another child and menopausal status which indicates state of their childbearing status.

**Table 6.** Test of significance of individual parameters of the Multinomial Logistic Regression Model.

Likelihood Ratio Tests				
Effect	Model Fitting Criteria	Likelihood Ratio Tests		
	−2 Log Likelihood of Reduced Model	Chi-Square	df	Sig.
Intercept	230.020	0	0	.
Age of women	233.704	3.684	2	0.158
Marital Status	234.244	4.224	2	0.121
Educational Qualification	234.405	4.385	3	0.223
Employment status	230.027	0.007	1	0.935
Menopausal Status	245.933	15.913	1	0.000
District	233.763	3.743	1	0.053
Desire another child	240.632	10.612	1	0.001

The model fitted in the analyses (Table 7) reveals that for the older adults who responded ‘Yes’ to the question, ‘Do you use FP to prevent pregnancy’, compared with those whose response was, ‘No’, older adult women who said they desired another child were significantly (*p* < 0.01) 7.5 times more likely to use family planning (FP) methods than those who said they do not desire another child. This result is interesting because it simply reveals that although they desired another child they still needed to use FP methods for other purposes such as to delay pregnancy and prevent infection of sexually transmitted diseases (STDs) and HIV. The probability of using FP methods by this group is also very high (0.88).

**Table 7.** Multinomial Logistic Regression Model.

Using contraceptive to stop pregnancy								95% Confidence Interval for Exp (B)		
Reference category: No		B	Std. Error	Wald	df	Sig.	Exp (B)	Lower Bound	Upper Bound	Probability of using FP method
Yes	Intercept	0.74	0.58	1.66	1	0.20				
Age	50–59	0.48	0.38	1.56	1	0.21	1.62	0.76	3.43	0.62
	60–69	−0.13	0.40	0.10	1	0.75	0.88	0.40	1.94	0.47
	70 and above	0.00	.	.	0	.	.	.	.	
Marital Status	Single	−0.60	0.32	3.56	1	0.06	0.55	0.29	1.02	0.35
	Married	−0.48	0.31	2.44	1	0.12	0.62	0.34	1.13	0.38
	Divorced/widowed/separated	0.00	.	.	0	.	.	.	.	
Educational Status	None	−0.76	0.38	3.91	1	0.05	0.47	0.22	0.99	0.32
	Primary	−0.48	0.39	1.51	1	0.22	0.62	0.29	1.34	0.38
	Secondary	−0.26	0.42	0.38	1	0.54	0.77	0.34	1.75	0.44
	Diploma/Degree	0.00	.	.	0	.	.	.	.	
Employment Status	Unemployed	0.02	0.29	0.01	1	0.94	1.02	0.58	1.81	0.51
	Employed	0.00	.	.	0	.	.	.	.	
Menopausal Status	Postmenopausal	−1.35	0.34	15.68	1	0.00	0.26	0.13	0.51	0.20
	Premenopausal	0.00	.	.	0	.	.	.	.	
District	Urban	−0.50	0.26	3.68	1	0.06	0.61	0.36	1.01	0.38
	Rural	0.00	.	.	0	.	.	.	.	
Desire another child	Yes	2.02	0.66	9.44	1	0.00	7.51	2.08	27.15	0.88
	No	0	.	.	0	.	.	.	.	

The older adult women who were in the postmenopausal state were less likely to use FP methods than those in their premenopausal state (OR = 0.13) with a probability of usage of FP method low and equal to 0.22. Older adult women from urban districts were less likely to use FP methods than those in the rural districts (OR=0.36). The probability of use of FP methods to prevent pregnancy increased with increased educational qualification. For stance, those with no schooling, had primary or secondary education were less likely to use FP methods than those with degree/professional qualifications. The probability of using FP methods by those with no schooling, primary or secondary education was respectively 0.32, 0.38 and 0.44. The single (never married) women were less likely (OR = 0.55) to use FP methods than the other marital status with probability of using FP equals 0.35. The married were also less likely to use FP methods than the other marital status with probability of use of FP methods equal to 0.38. The unemployed older adults were 1.02 times more likely to use FP methods than the unemployed with probability of use of FP methods as 0.51. The older adult women aged 50–59 years were respectively 1.62 times more likely to use FP methods than those aged 70 years and over with probability of using FP methods as high as 0.62 while those aged 60–69 years were less likely to use FP methods.

### Discussion of the results

This paper sets as its goal to analyse, at the individual level, how the socioeconomic and demographic factors such as age of older adults’ women, marital status, highest educational qualification, employment status, menopausal status, desire another child and location are associated with their use of family planning methods and how these socio-economic and demographic characteristics influence their family planning use. The study showed that 53.2% of the older adults were between 50 and 59 years while 42.8% had no schooling; 32.9% were married and 68% unemployed. These results have strong implications on the utilisation of family planning for preventing/delaying future pregnancy as well as preventing STI and HIV infection.

The continued engagement of 35% of the studied sample in sexual activity with their partners implies that the women are vulnerable to unwanted pregnancy, as well as STI and HIV infections, if they do not use family planning methods. This result provides further motivation for studying the family planning use by the older adults. WHO (1996) reported that no accurate biological marker exists that truly defines the moment when fertility ceases. Furthermore, this result is consistent with Patel et al. (2003), Koster and Garde (1993), Koster (1991) and OlaOlorun (2013) who have shown that women, 50 years and over, are still sexually active and engage in unprotected sex, thinking that they can no longer be pregnant. There is therefore need for older adults to continue the use of family planning services even after attaining menopause so long as they are sexually active.

The study shows that 42.8% of the older adult women had no schooling. It is not unlikely that as girls, they were denied the opportunity to go to school or dropped out before completing their education. This result has strong implications on the utilisation of family planning by the older adults. It has been reported (UNFPA and HelpAge International, 2012) that in developing countries, an average of 58 per cent of women aged 65 or over is illiterate, compared with 34 per cent of men in the same age group. The low level of educational qualification of the sampled group has implication on the level of usage of family planning methods as most of the available methods are explained in English and they would therefore require an interpreter to facilitate the understanding of the directions for usage. It can seriously limit their ability to obtain information and access services (de Vaus, Gray, & Stanton, 2003). Little wonder why a little over one in every four (25.2%) of the older adult women was still using FP methods, notwithstanding the high rate of HIV infection in Botswana (19.03%) (BAIS IV, 2013) and STIs (7.1%) (Tafuma et al., 2014). It is important that efforts be made to improve the literacy levels of older women (de Vaus et al., 2003).

This study shows that FP use among the older adults increases with increased educational qualification. For instance, older adults with no education are less likely to use FP planning methods than those with secondary education qualifications. This result does not come as much of a surprise as higher educational attainment increases female decision-making power and awareness of the benefits of good family planning practices (Stephenson & Hennink, 2004). Education is an indicator of income level; the more educated women are, the most likely they are to be employed in better paid jobs and earn more money. They are able to pay for contraceptive services of their choice than those with no education. The result is consistent with findings of Radulović, Šagrić, Višnjić, Tasić, and Marković (2006), who in a study with women aged 15–49 years showed that the women with primary education use less protection from unwanted pregnancy than the women with secondary and higher education. Thus, while 66.7% of the women with primary education use some method of protection from unwanted pregnancy, the corresponding percentage of the women with secondary education and the women with higher level of education are, respectively, 82.3% and 84.6% (see also Eliason, Awoonor-Williams, Eliason, Nonvignon, & Aikins, 2014). Education also has been observed in many other studies to be associated with contraceptive use (Arbab et al., 2011; Faisal & Eria, 2014; Jabeen, Gul, Wazil, & Javed, 2011; Yihunie et al., 2013). Women with at least primary level of education are 8-10% more likely to be using a modern, or any method of contraception compared to those with no education. Yihunie et al. (2013) found that educated women had better odds of using modern contraceptive methods than uneducated married women. Improved education would therefore be essential to the older adults to facilitate their knowledge of appropriate FP methods to use.

The menopausal status of older adult women is negatively moderately correlated with a desire to use FP methods to prevent further pregnancy. For example, postmenopausal women (86.3%) are less likely to use FP methods than the premenopausal women (13.7%). The results also show that 13.7% of the older adult women who have not attained menopause are very susceptible to pregnancy. Use of FP methods to prevent future pregnancy is highly recommended since as noted by WHO (1996), it is possible for a woman to attain menopause and yet still be fertile. Age, in this study is shown to be negatively significantly correlated with FP use. Thus older adult women aged 50–59 years were found to be less likely to use FP methods than those aged 70 years and over. The likelihood to find a woman still desiring to have children and being sexually active is higher within the age group 50 - 59 than in the other age groups as many of them may not have attained menopause. The results of the studies by Jabeen et al. (2011) and Sharma, Mohan, Das, and Awasthi (2012) which revealed that use of family planning methods was found more among women of higher age group than those of lower age group agree with the findings in this study even though the studies were based on women of the sexual reproductive ages, 15–49 years. Also Yihunie et al. (2013) found out that age had an inverse association with use of modern contraceptive methods.

Marital status is not a significant predictor of family planning use. The results show that older adult women who are single (never married) were less likely to use FP than the married/divorced/widowed/separated but the probability of FP usage by the single women (0.35) is slightly lower than the married (0.38). Thus while those who are married use family planning when they want to delay/space out pregnancies, the single (never married) use it to prevent getting pregnant completely. Yihunie et al. (2013) found that older married women had lower odds of using modern contraceptive methods than younger married women. The single women are also more likely to be exposed to different partners and HIV as well as STIs. The study by Kposowa (2013) found out that while single (never married) persons were 13 times as likely to die of HIV/AIDS as their married counterparts, the divorced and separated individuals were 4.3 times more likely to die of HIV/AIDS than married individuals.

Location was shown in this study not to be a significant factor to family planning use by the older adults; however, urban dwellers are less likely to use FP methods than rural dwellers. Although Pebley, Goldman, and Rodriguez (1996) have shown that women in communities with stronger health service presence were more likely to seek reproductive health care services, women’s decisions to use family planning services are influenced by the accessibility of health services in the community and by the general socio-economic status of the community since stronger health infrastructure and higher socioeconomic status decrease logistical barriers to seeking services (Burgard & Lee-Rife, 2009). In addition, Yihunie et al. (2013) showed that married women who lived in rural areas had 30% lower odds of using modern contraceptives than urban married women, a contradiction to our results. Access to health care facilities in Botswana in terms of distance covered to health centres is not a problem as they are evenly spaced in both urban and rural areas and about 90% of people in Botswana are known to live within 15 kilometres of a health centre (Centre for Health and Gender Equity 2012). It is likely that older adult women in the rural areas are exposed to both the natural as well as the modern FP methods while those in the urban areas concentrate more on the modern methods.

Employment status of the older adults significantly negatively predicts family planning use. Thus, the unemployed are almost as likely to use FP methods as the employed. This result is not unexpected because healthcare services in Botswana are supplied almost free of charge to the local population. FP methods, where available, are also obtained free of charge from the healthcare services. The implication is that both the unemployed and employed can access the services equally. Little wonder why employment which is an indicator of income is not a significant predictor of FP use. This result, however, appears to contradict the results of the analysis of 10,204 women of reproductive ages 15–49 years from the 2011demographic and health survey data of Ethiopia (Yihunie et al., 2013) which reported that being employed increased the likelihood of using modern contraception because they are more able to pay for the services. The FP methods are obtained at no financial costs to the users in Botswana. For instance, condom is available freely at all strategic points (toilet ends, educational institutions, government offices, malls, etc.).

Desire to have another child is shown in this study to be a highly significant (*p* < 0.01) predictor of FP methods use. Thus, older adults who desire to have another child are 7.5 times more likely to use FP methods than those who do not desire another child. It would have been expected that the women who desire another child would not need FP methods but the implication of this result is that they still need to use FP methods for other purposes such as to delay pregnancy or prevent infection of sexually transmitted diseases (STDs) and HIV. This finding is supported by the results of the 2013 National Survey of Sexual Attitudes and Lifestyles (NATSAL) research project (Wellings et al., 2013) which showed that 1 in 5 pregnancies conceived when the mother is aged 40 years or older are unplanned and 28% of these pregnancies end in termination. Also in the Western world, in women over the age of 40 years, it is shown (Rao & Demaris, 1995) that relationship breakdown easily and re-partnering is on the increase, while sexual intercourse occurs more frequently in the new relationships. The result of these new relationships and increases in sexual frequencies is that sexually transmitted infection (STI) rates are increasing most rapidly (Public Health England, 2015). The older adults’ women, therefore, need interventions that will assist them manage their later lives effectively. Family planning services and information need to be provided to them freely.

## Conclusions

As has been argued in this paper, the family planning needs of the older adults are totally neglected, yet many of them, who are still in their early menopausal state, are still sexually active and prone to unwanted pregnancy, HIV and STIs. There is a need for the healthcare managers and policy makers to address these needs in the healthcare systems.

Education has been shown to be a major contributory factor to FP use. Unfortunately, a substantial percentage of the older adult women do not have the requisite education and literacy level to enhance understanding of the utility of family planning. The paper therefore recommends the need to consider raising the levels of formal education of the older adults. Promoting basic education, especially among females, will be a crucial step. Education that is age sensitive and directed towards family planning services and use is of utmost importance.

The high probability of FP use by women who though over 50 but desire another child calls for the urgent need for Public Health Department of the Ministry of Health to come up with interventions which will be home based, considering the age of the women, to assist the older adults meet their FP needs. Training in the varieties of available modern and natural FP methods is of utmost importance to enable them make right choices of methods to use.
